# Ultra‐Flexible Pixelated Perovskite Photodetectors Enabled by Honeycomb Polymer Grids for High‐Resolution Imaging

**DOI:** 10.1002/adma.202415068

**Published:** 2025-03-17

**Authors:** Ding Zheng, Zhaoqian Xie, Wei Huang, Dongjun Bai, Jaehyun Kim, Dan Zhao, Fei Qin, Dayong Zhang, Joon‐Seok Kim, Jianhua Chen, Yao Yao, Zhi Wang, Sharma Sakshi, Juan‐Pablo Correa‐Baena, Lincoln J. Lauhon, Mercouri G Kanatzidis, Tobin J. Marks, Antonio Facchetti

**Affiliations:** ^1^ Department of Chemistry and the Materials Research Center Northwestern University Evanston IL 60208 USA; ^2^ State Key Laboratory of Structural Analysis Optimization and CAE Software for Industrial Equipment Dalian University of Technology Dalian 116024 P. R China; ^3^ Department of Engineering Mechanics Dalian University of Technology Dalian 116024 P. R China; ^4^ DUT‐BSU Joint Institute Dalian University of Technology Dalian 116024 P. R China; ^5^ Department of Materials Science and Engineering Northwestern University Evanston IL 60208 USA; ^6^ School of Materials Science and Engineering Georgia Institute of Technology Atlanta GA 30332 USA

**Keywords:** flexible, high resolution, perovskite, photodetector, photodiode

## Abstract

A nature‐inspired fabrication method based on a photolithography‐free flexible polymer grid is reported for high‐resolution pixelation of perovskite photodiode arrays with exceptional mechanical ductility and a morphology resembling that of natural compound eyes. The resulting pixelated perovskite photosensitive layer has a ≈1 µm pixel size with 2000 Pixels per inch (PPI) resolution when fully assembled as a photodetector array, delivering a detectivity of >10^13^ Jones while providing cross‐talk free imaging. Using a polymer grid effectively releases stress on the perovskite platform, greatly increasing the mechanical agility of the otherwise brittle perovskite film. This novel fabrication methodology and device design offer new possibilities for applications in robotics, biomedical imaging, and virtual and augmented reality.

## Graphical abstract

Nature‐inspired, photolithography‐free, flexible, pixelated perovskite photosensitive films serve not only as a high‐resolution pixelation platform but also as a stress‐relief mechanism for otherwise brittle perovskite films, enabling enhanced mechanical flexibility. This synergetic approach yields pixelated perovskite layers with ≈1.5 µm pixel sizes, ≈100 nm gaps, and 2000 PPI resolution. Assembled photodetector arrays offer detectivity greater than 10^13^ Jones with cross‐talk‐free imaging. The flexible polymer grid also enhances mechanical durability, expanding potential applications in robotics, biomedical imaging, and virtual/augmented reality.

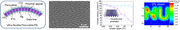

## 1 Introduction

**Figure 1:**
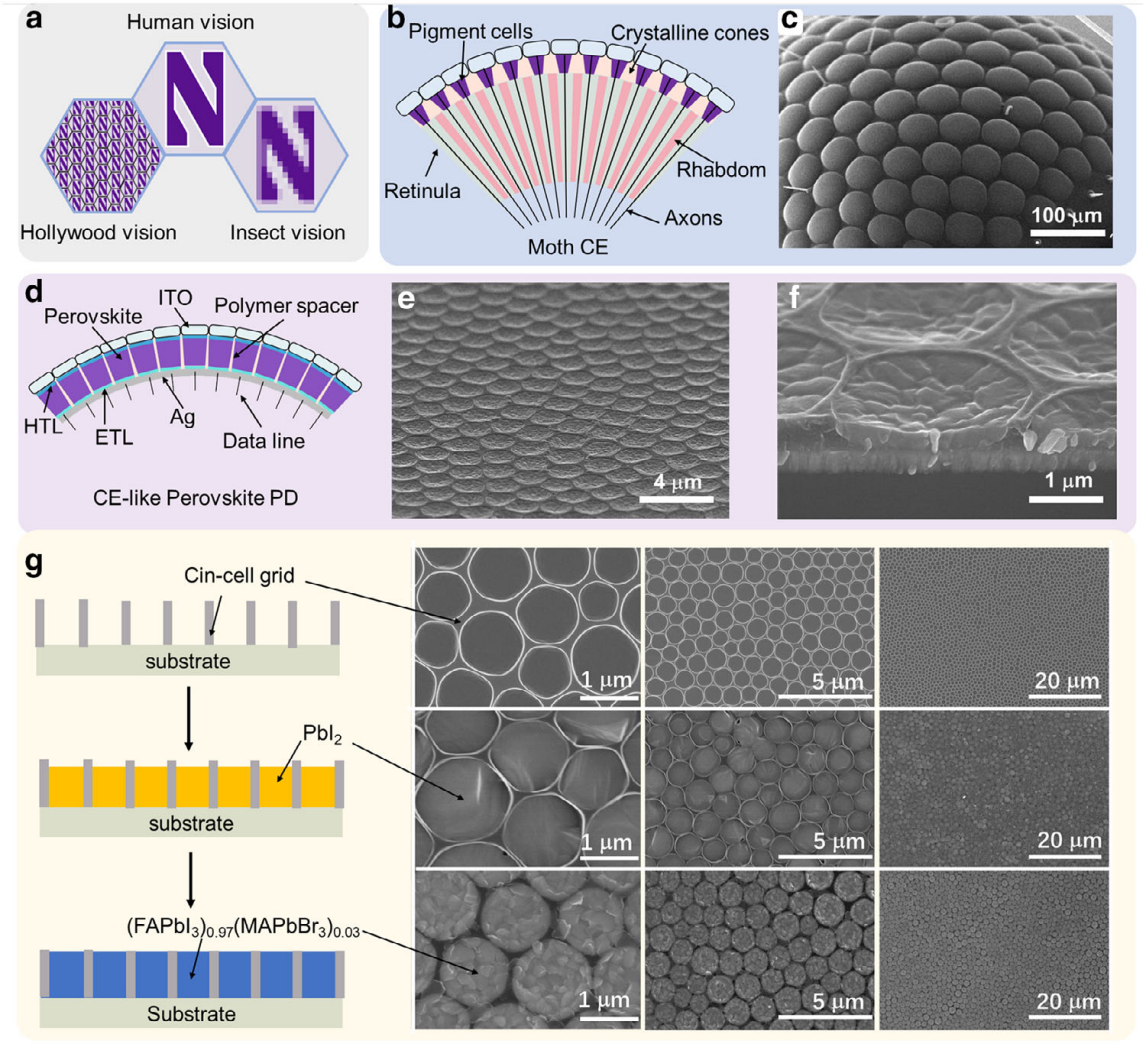
Types of vision, pixelated perovskite films, and structure/fabrication of a Perovskite PDs array of this study. a) Schematic representation of three different vision modalities (Hollywood, human, and insect) showing that insect vision is similar to the human but with lower resolution. b) Insect CE structure and c) SEM image of a real moth eye. d) pixelated perovskite PD structure created in this study and e,f) SEM image of a pixelated perovskite layer. g) Fabrication steps for embedding/pixelating the perovskite material into a polymer grid, affording pixelated perovskite films (left) and corresponding SEM images (right).

Natural visual sensors are mainly composed of two groups: single‐lens eyes, such as the human eye, and compound eyes as in arthropod insects (**Figure** **1**a).^[^ ^1^ ^]^ While single‐lens eyes (SLEs) have the advantages of high‐light efficiency and spatial resolution, compound eyes (CEs) outperform them in terms of motion detection and a wider field of view. However, compared with SLE, compound eyes have never been widely adopted for abiotic optical instrumentation because of limited spatial resolution, complex processing requirements for hemispheric devices, and the requirement of high integration levels in micro‐devices.^[^ ^1^, ^2^, ^3^ ^]^ Thus, instead of realizing compound eye vision, several pioneering research groups have integrated micro‐lens arrays with single imagers, yielding a vision system commonly known as “Hollywood vision” (Figure 1a).^[^ ^4^, ^5^ ^]^ CEs consist of several small individual eye elements, namely ommatidia, each having an independent sensory nerve enabling faster light response than SLEs (Figure 1b).^[^ ^6^, ^7^ ^]^ Furthermore, CEs have a spherical surface with a large curvature (Figure 1c), providing a wide field of vision in all directions. In some embodiments, a pair of CEs can measure range and velocity by acting as a photoelectric radar^[^ ^7^ ^]^ and could be an essential component in future micro‐robotic, man‐machine interactions, or medical endoscopy technologies.^[^ ^2^, ^8^, ^9^, ^10^, ^11^, ^12^ ^]^ While previous studies have demonstrated artificial photodetector (PD) devices incorporating CE systems (Table S1, Supporting Information), they were only in rigid PDs and/or in large‐area/low‐resolution platforms with limited image quality.^[^ ^13^, ^14^, ^15^ ^]^ Here, we demonstrate a bionic perovskite PD using a solution‐processed microscopic polymer grid to pixelate photosensitive perovskite films with exceptional resolution, respectable detectivity, and absence of cross‐talk. Using these micro‐diode perovskite PD elements, a flexible imager is demonstrated on a plastic substrate that can be laminated to hemispheric elastomeric supports with varying curvatures, enabling high‐speed and adjustable wide‐angle sensing.

## 2 Results and Discussion

### 2.1 Fabrication of Pixelated Perovskite Films

The schematic representation and scanning electron microscopic (SEM) images of the different fabrication stages affording the pixelated perovskite photosensitive array within a polymer grid are shown in Figure 1d, and fabrication details can be found in the Supporting Information (Figures S1–S5, Supporting Information). Briefly, the polymer grid is fabricated on a sacrificial substrate using the breath figure patterning method (Figure S1, Supporting Information),^[^ ^16^, ^17^, ^18^, ^19^ ^]^ which consists of spin‐coating a photocurable cinnamate‐cellulose polymer solution (polymer = Cin‐Cell, see structure in Figure S1a, Supporting Information) in a high‐humidity atmosphere (relative humidity >90%), UV crosslinking, and Cin‐Cell grid transfer to the desired platform (Figure S1b, Supporting Information; Figure 1g‐top). Next, for perovskite pixelation, a PbI_2_ solution is spin‐coated onto the polymer grid, and, after annealing at 70 °C, the dried PbI_2_ is confined within the polymer grid walls (Figure 1g‐center). Finally, a FAI:MABr:MACl solution was spin‐coated on top, and the resulting film was annealed at 140 °C, affording a pixelated perovskite film of composition (FAPbI_3_)_0.97_(MAPbBr_3_)_0.03_ (Figure 1g‐bottom).^[^ ^20^, ^21^, ^22^, ^23^ ^]^ The properties of the unpatterned/pixelated perovskite films were accessed by multiple characterization techniques, including steady‐state optical absorption and transmittance, X‐ray diffraction (XRD), photoluminescence (PL), and X‐ray photoelectron spectroscopy (XPS) measurements (Figures S6–S10, Supporting Information). The results demonstrate that the perovskite structure and phase formation are consistent between the unpatterned and pixelated films, confirming a uniform distribution of the halide composition across both types of films. This uniformity supports the conclusion that the perovskite material is homogeneous without detectable phase separation or compositional variation between distinct regions of the films.^[^ ^24^, ^25^ ^]^ Each perovskite pixel is 1.54 ± 0.29 µm in diameter and separated by 146 ± 28 nm thick polymer walls, thereby affording an array with a resolution of ≈16500 Pixels per inch (PPI), which represents the upper‐resolution limit assuming the electrode size matches the perovskite grain sizes. (Figure S11, Supporting Information). To demonstrate that the Cin: Cell grid is effective in suppressing cross‐talk in pixelated perovskite films, conducting AFM (c‐AFM) measurements were carried out on the perovskite layer without (unpatterned perovskite film) and with the polymer grid (pixelated perovskite film), and using ITO as the transparent electrode (see Figure S12, Supporting Information). Due to the considerable charge diffusion length (≈2 um) of metal halide perovskites,^[^ ^22^, ^26^, ^27^, ^28^, ^29^ ^]^ the unpatterned film under both weak and strong illumination conditions exhibits significant photocurrent in the unexposed areas. This results from significant cross‐talk, which will inevitably lead to a blurred image for devices aiming for resolutions greater than ≈1000 PPI.^[^ ^30^, ^31^, ^32^ ^]^ However, for the pixelated perovskite films, no photocurrent is detected in the polymer region or the unexposed perovskite pixels near the illuminated regions, demonstrating the ability of this pixelation method to achieve high‐resolution devices.

### 2.2 Perovskite Photodiode Array

**Figure 2:**
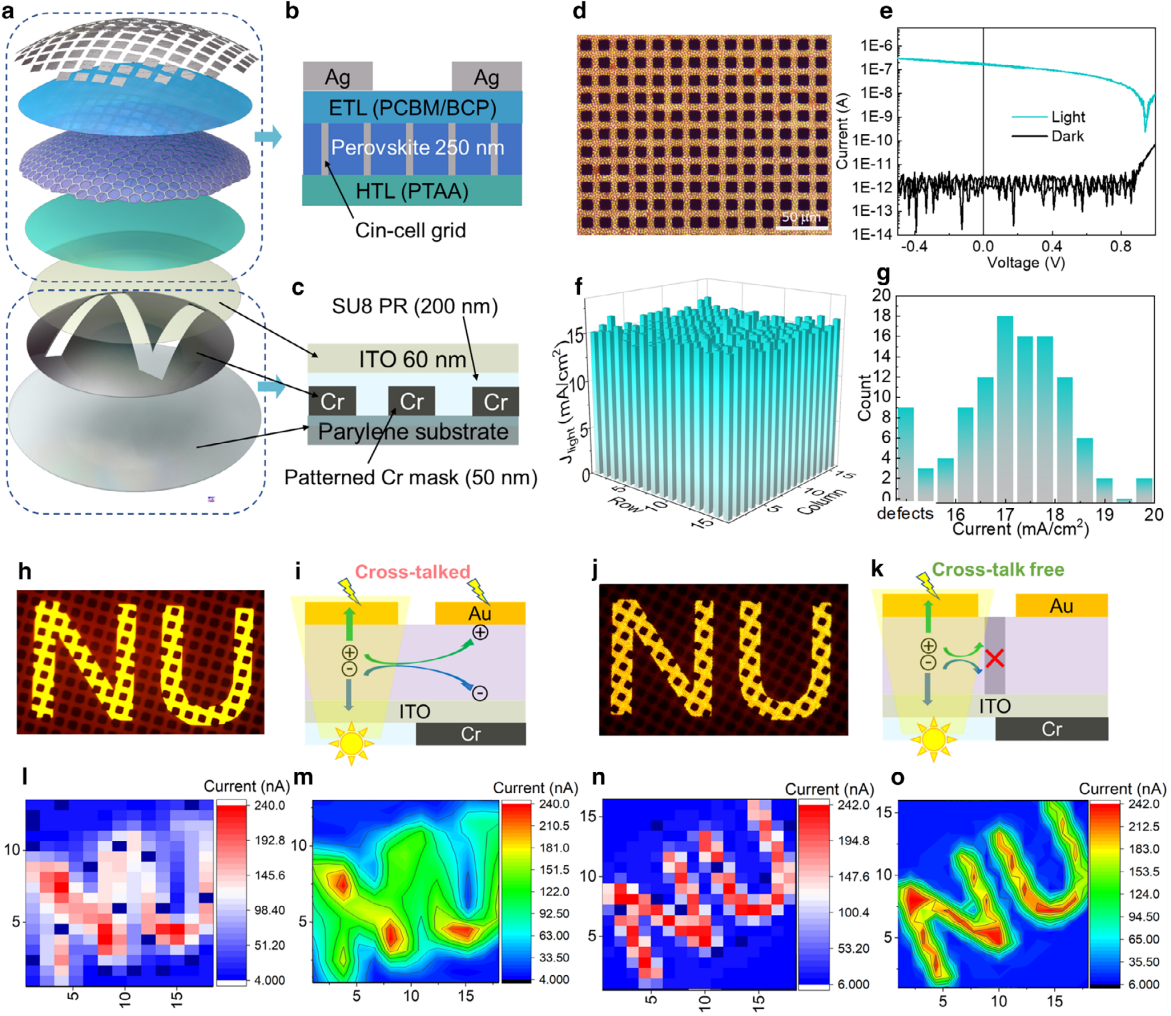
Device structure and performance of 2000 PPI arrays. a–c) Device architecture of a 2000 PPI perovskite photodiode array with the integrated shadow mask highlighted in (c). d) Optical image of a perovskite photodiode array (scale bar = 50 µm). e) Current under AM 1.5 G illumination and in the dark of an individual pixel of the perovskite photodiode array. f) Light current density distribution of a 2000 PPI photodiode array. g) Statistical distribution histograms of the light current density for 256 pixels, including 9 with defects. h) Microscope image of an unpatterned perovskite photodiode array with the “NU” pattern illuminated. i) Schematic representation of cross‐talk in the unpatterned perovskite film. j) Microscope image of a pixelated perovskite device with the “NU” pattern illuminated. k) Schematic representation of cross‐talk suppression in a pixelated film. l–o) Current mapping and contour current mapping of an unpatterned perovskite l,m) and pixelated n,o) perovskite photodiode array under 100 mW cm^−^^2^ white LED.

Next, to further verify the absence of cross‐talk, a fully functional and ultra‐flexible perovskite Photodiode Array based on the pixelated perovskite film was fabricated on parylene, and the performance was compared to the device using an unpatterned perovskite film (**Figure** **2**a,b). These photodetector devices were prepared on a parylene substrate on which, optionally, an integrated chromium photomask (<100 µm) with a patterned NU image was deposited by thermal evaporation, planarized with SU8, and then coated with sputtered ITO (Figure 2c). On top of the ITO film a hole transporting layer (HTL, PTAA) was deposited, followed by lamination of the pixelated perovskite film, an electron transporting layer (ETL, PCBM: PMMA/BCP), and finally, to realize high‐resolution imaging, the device was completed by depositing an Ag electrode array (12.5 µm × 12.5 µm) fabricated by thermal evaporation of Ag through a 2000 mesh TEM grid functioning as a shadow mask (Figure 2d). For the device with the unpatterned perovskite film, the fabrication procedure was identical, with the exception that the perovskite film was deposited by a conventional method (see detail in Supporting Information). Both devices have a resolution of 2000 PPI, dictated by the TEM mask size. The representative *I*–*V* curves in the dark and under illumination and with a statistical distribution of the photocurrent density for all devices are shown in Figure 2e–g (pixelated) and Figure S13 (Supporting Information) (unpatterned). Due to the ultra‐small dimension of these pixels, the dark current is below the sensitivity of our instrumentation (<1 pA), however, for an array of 256 pixels, the average photocurrent density achieved is 15.41 ± 0.71 mA cm^−2^ with a working device yield >96%. Next, we assessed the photocurrent mapping of the photodetector devices with the NU letter mask (Figure 2h–o), demonstrating that the present pixelated perovskite device exhibits a sharper and more recognizable image than the unpatterned perovskite control device due to the absence of cross‐talk.

Furthermore, we extracted current data from five pixels for both the unpatterned and pixelated devices, as shown in Figure S14 (Supporting Information). The results indicate that the pixelated devices exhibit significantly lower dark current at locations 2 and 3, which correspond to the non‐illuminated pixels, demonstrating that the pixelated devices reduce the leakage current between pixels, thereby mitigating cross‐talk effects.

### 2.3 Perovskite PDs Array

**Figure 3:**
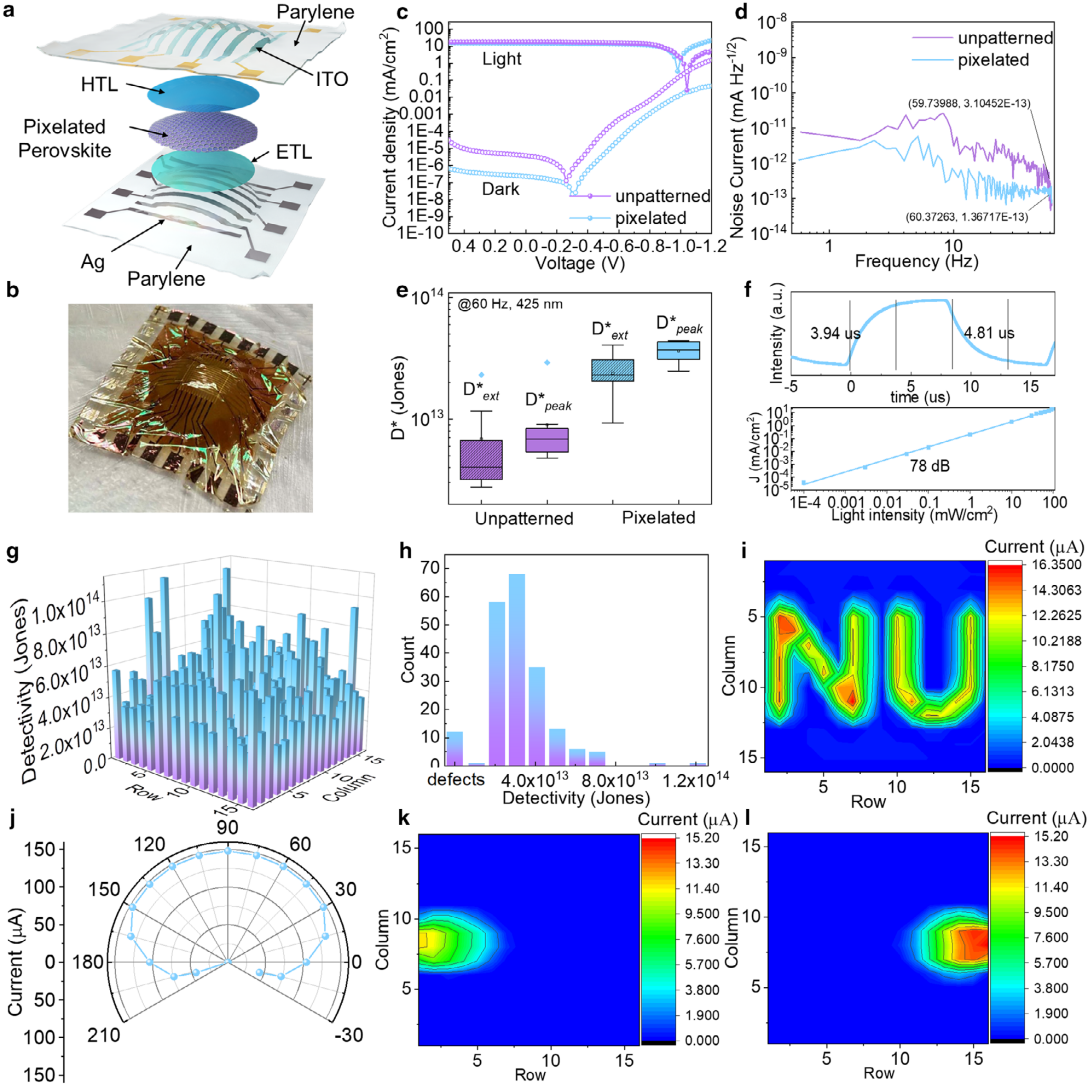
Performance of a 16 × 16 cross‐bar ultra‐flexible perovskite PD array. a) Device architecture of a 16 × 16 cross‐bar perovskite PD array. b) Photograph of a perovskite PD array conformed to a hemispherical support. c) Light current under AM 1.5 G and dark current of single device pixels of unpatterned and pixelated perovskite PDs which conformed on a hemispherical support. d) Noise current measurements of unpatterned and pixelated perovskite PDs. e) Comparison of D*_peak_ and D*_ext_ of unpatterned and pixelated perovskite PDs. f) Response transit time (top) and dependence of the photocurrent density on the illumination intensity range from 0.0001 to 100 mW cm^−2^ (bottom) of an individual pixel of a pixelated perovskite PD. g–i) Detectivity mapping (g), detectivity statistical distribution histograms including 12 defects (h) and contour current mapping (i) of a 16 × 16 cross‐bar pixelated perovskite PDs (under 100 mW cm^−^^2^ white LED). j) Incident angle‐dependent light current of a 16 × 16 cross‐bar pixelated perovskite PD mounted on a hemispherical support. Contour current mapping of a laser spot with an incident angle of k) −18 and l) 198 ^o^ under 85 mW cm^−^^2^ 532 nm laser beam_._

To evaluate the photodiode performance following more standard protocols, 16 × 16 cross‐bar PDs array with unpatterned and pixelated perovskite photosensitive films were fabricated. These devices have top strip metal electrodes (100 µm × 1 cm), which were patterned by a shadow mask, and strip bottom ITO contacts (1 cm × 100 µm) patterned by photolithography, yielding a 100 µm × 100 µm crisscross‐area (Figure S15, Supporting Information). All PD array devices were fabricated on 4 µm thick parylene substrates and encapsulated with a 4 µm parylene film on top. Finally, the PD array devices were laminated to hemispherical supports to mimic the curvature of compound eyes (**Figure** **3**a,b; Figure S16, Supporting Information). Representative light (AM 1.5 G) and dark current densities are plotted in Figure 3c; additional metrics are shown in Figure S17 (Supporting Information). Current–voltage measurements indicate that the photocurrent of the patterned photodetector device is slightly lower than that based on the unpatterned film (14.76 ± 1.83 mA cm^−2^ vs 16.32 ± 2.51 mA cm^−2^ @ −0.3 V), a result expected since the polymer grid is an electrical insulator and occupies ≈20% of the photodiode area (see calculation in the Supporting Information). However, the dark current density of the photodetector device is ≈ 10 × lower [1.24 ± 1.86 × 10^−7^ mA cm^−2^ (unpatterned) versus 3.71 ± 2.34 × 10^−8^ mA cm^−2^ (patterned)] due to efficient high‐resolution perovskite film patterning.^[^ ^33^, ^34^, ^35^ ^]^ Due to the decreased dark current, the peak detectivity (D*_peak_) of the patterned PD device array is significantly greater (3.91 ± 1.41 × 10^13^ Jones) than that of the unpatterned PD device (5.66 ± 1.41 × 10^12^ Jones) (Figure S18, Supporting Information). Note D*_peak_ of both PD devices is consistent with the extrapolated detectivity (D*_ext_) obtained from noise current measurements [6.84 ± 6.31 × 10^12^ Jones (unpatterned, 2.38 ± 0.92 × 10^13^ Jones (patterned)] (Figure 3d,e). These detectivities not only exceed those of state‐of‐the‐art perovskite PDs (10^12‐13^ Jones) but also exceed that of industry‐standard silicon PDs (10^12^ Jones) due to the constrained charge transfer in the dark.^[^ ^36^, ^37^, ^38^ ^]^ Figure 3f‐top shows the dependence of the photocurrent and current density on the illumination intensity ranging from 0.0001 to 100 mW cm^−2^. The photocurrent can be fit to a quasi‐linear power‐law relationship, resulting in a large linear dynamic range (LDR) of 78 ± dB.^[^ ^39^, ^40^ ^]^ The perovskite PD turn‐on transient time is 3.94 µs, and the turn‐off transient time is 4.81 µs (Figure 3f‐bottom), values that could be increased when connected to an adaptive drive circuit.^[^ ^41^, ^42^ ^]^ The construction of integrated circuits requires that the PD arrays as imaging units be fabricated with greater uniformity and higher yields.^[^ ^43^ ^]^ We therefore measured the *J*–*V* characteristics of the Pervoskite PD's 256 pixels and achieved a respectable device yield of >95% and a narrow distribution of D*_peak_ of 3.63 ± 1.51 × 10^13^ Jones (Figure 3g,h; Figures S19 and S20, Supporting Information). Note that our cleanroom is a class >1000, and key fabrication processes, e.g., ITO sputtering and parylene deposition, were carried outside the cleanroom; thus, state‐of‐the‐art yields cannot be expected. Next, device photocurrent mapping (Figure 3i) and field of view (FOV) (Figure 3j) were also assessed. FOV measurements are conducted with a custom‐made holder and turntable to precisely adjust the incident angle of the laser beam (Figure S21, Supporting Information). Thus, the angle‐dependent light current of this device indicates that the maximum visual field (except the blind area) is ≈216°, whereas that of the corresponding planar device is only ≈179° (Figure S22, Supporting Information). The current mapping of a laser spot at −18° and 198° incident angles is presented in Figure 3k,i, demonstrating that the light pattern is recognizable and well‐imaged by the present hemispherical perovskite PD device. To investigate the device response under realistic environmental conditions, we measured for the encapsulated devices, the on‐off operation stability (Figure S23, Supporting Information), and light soaking stability (Figure S24, Supporting Information) under the stress conditions of T = 25 °C and RH = 45%. The results show that the perovskite PD device maintains a stable turn‐on current after 28800 s of operation at a 2 Hz switching frequency. In the long‐term stability test, unencapsulated devices were aged in a chamber under continuous white LED exposure at an intensity of 100 mW cm^−^^2^. After 10 days of light exposure, the light current retained >80% of the initial value, demonstrating good light stability, which will certainly improve upon encapsulation.

### 2.4 Photodetector Response Upon Mechanical Deformations

**Figure 4:**
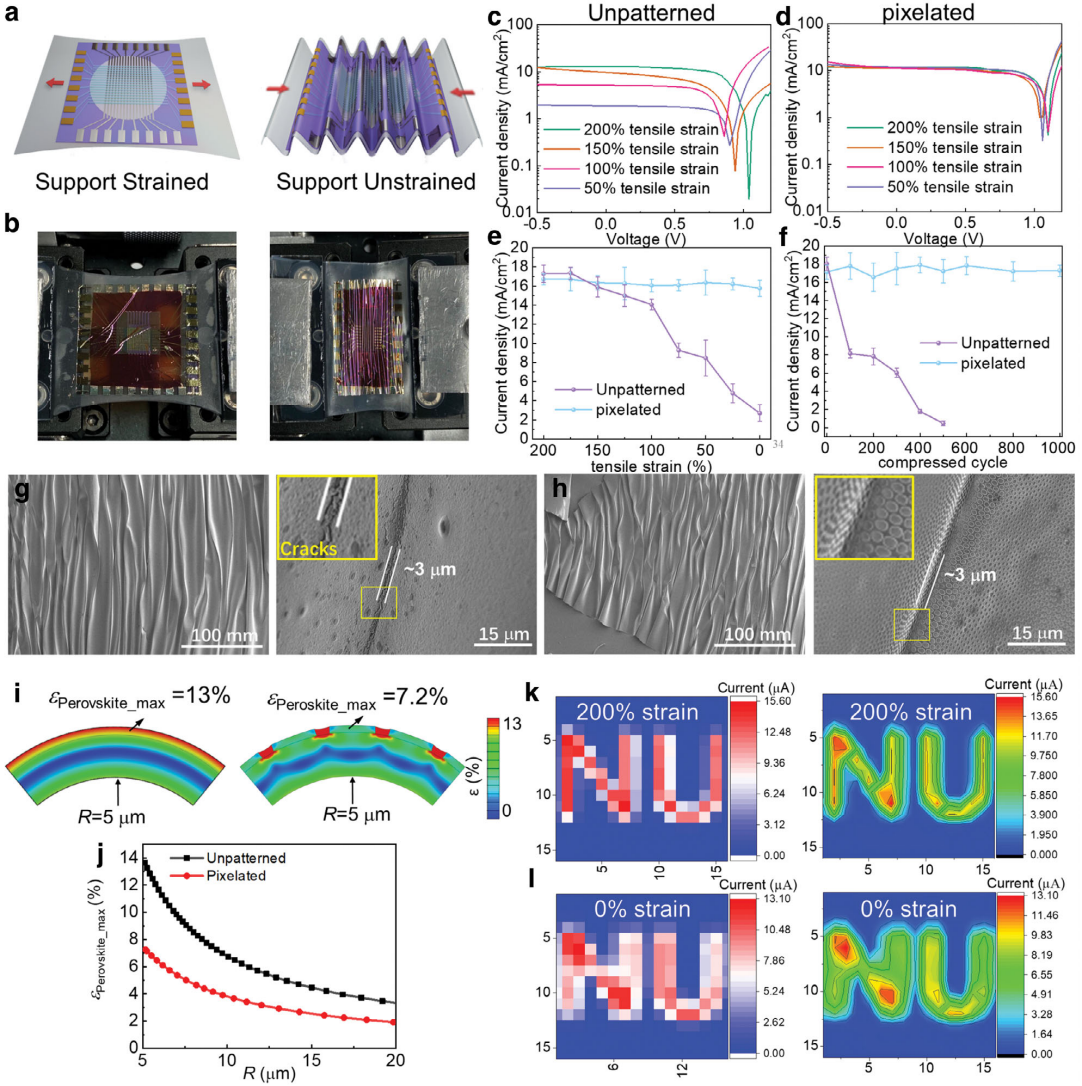
Flexibility of unpatterned and pixelated perovskite PDs. a) Schematic representation of the flexibility test for perovskite PDs laminated on a pre‐stretched elastomeric support. b) Photograph of a perovskite PD device under mechanical deformation. *J*–*V* curves of c) unpatterned and d) pixelated perovskite PDs under AM 1.5 G illumination for different tensile strains. e) A light current density of unpatterned and (d) pixelated perovskite PDs with different tensile strains. f) A light current density of unpatterned and pixelated perovskite PDs after different strain cycles. SEM image of g) unpatterned and h) pixelated perovskite films under 0% tensile strain. i) The distributions of maximum principal strain ɛ under pure bending of composite films (a) and (b) with bending radius R = 5 µm, where ɛ_Perovskite_max_ is the maximum ɛ in Perovskite material. j) The maximum principal strain in the unpatterned perovskite layer and pixelated perovskite under pure bending. Current mapping and contour current mapping of pixelated perovskite PDs under strained k) and unstrained l) under 100mW cm^−2^ white LED.

Finally, the performance of the unpatterned and pixelated perovskite PDs upon mechanical deformation at different strains was also evaluated. For this experiment, the perovskite PDs were delaminated from the supporting glass (Figure S16, Supporting Information) followed by lamination to a 200% pre‐stretched elastomeric support (VHB tape, 3m), and finally, the strain to the support was released. **Figure** **4**a,b display schematic representations and photographs of a flexible perovskite PD subjected to the tensional test, respectively.^[^ ^44^, ^45^, ^46^, ^47^ ^]^ Thus, when the strain on the support varies from 200% to 0%, the photocurrent density of the unpatterned device decreases from 17.61 ± 1.31 to 3.23 ± 0.18 mA cm^−2^, corresponding to a reduction of more than 82% (Figure 4c,e). In contrast, the pixelated perovskite PD exhibits negligible photocurrent variation (16.93 ± 0.84 mA cm^−2^ at 200% strain and 16.35 ± 0.55 mA cm^−2^ at 0% strain) (Figure 4d,e). This result indicates that the softer pixelated perovskite film, containing the Cin‐Cell polymer (elastic modulus of ≈0.6 GPa), can efficiently release the strain on the otherwise brittle perovskite materials (10–12 GPa),^[^ ^27^, ^48^, ^49^, ^50^ ^]^ resulting in negligible electrical performance degradation. In addition, upon repetitive strain cycles (substrate strain 200% ↔ 170%) the photocurrent of the unpatterned device falls by ≈ 50% (from 17.72 ± 1.24 to 1.03 ± 0.74 mA cm^−2^) after ≈ 100 cycles and the device ceases to function at ≈ 500 cycles while that of the pixelated perovskite PD decreases slightly from 17.44 ± 0.45 to 16.57 ± 0.82 mA cm^−2^ after 1000 cycles (Figure 4f). Additional substantiations of the enhanced elasticity of the pixelated versus unpatterned PDs come from analyzing the film topology and simulated mechanical behavior of the corresponding pixelated and unpatterned perovskite films when laminated/stressed on the elastomeric substrate. Thus, SEM imaging shows the formation of high‐aspect‐ratio sinusoidal wrinkles with a bending radius as low as ≈3 µm when the strain to the support is released (Figure 4g,h). However, the unpatterned perovskite film exhibits severe crack formation (Figure 4g), and no significant cracks are detected in the pixelated film (Figure 4h). In Furthermore, finite element analysis (FEA) simulations of the film‐on‐elastomer demonstrate that perovskite pixelation with a polymer matrix significantly enhances ductility under strain (Figure 4i; Figure S25, Supporting Information). Thus, when the films are bent to a 5 µm radius, the maximum strain (ɛ_max_) acting on the perovskite area of the pixelated film is only 7.2%, which is 44% lower than that accumulated in the unpatterned perovskite film (ɛ_max_ = 13%) (Figure 4j). Clearly, for the pixelated film, most of the strain is localized in the organic polymer matrix, which can better tolerate stress than a crystalline inorganic film, fully explaining the experimental tensile behavior of these films and PDs.

## 3 Conclusion and Outlook

In summary, this work demonstrates a Nature‐inspired fabrication of high‐performance perovskite PDs having exceptional mechanical flexibility enabled by a pixelated perovskite film having a morphology and structure like that of CEs. For the first time to our knowledge, the mechanical ductility of brittle perovskite‐based devices photodetector has been elevated to the same level as organic and polymer optoelectronic photodetectors.^[^ ^40^, ^41^, ^42^, ^51^, ^52^, ^53^ ^]^ The breath Figure fabricated polymer grid acts as an effective stress release component, greatly enhancing mechanical energy dissipation of the otherwise brittle perovskite film. The new devices exhibit superior detectivity of >10^13^ Jones and a resolution of 2000 PPI, which may have the potential to extend more than 16 500 PPI by electrode engineering in the future. This novel fabrication method and device architecture opens new possibilities for systems designs in diverse applications and should enable the integration of high‐performance inorganic materials in a flexible form to fabricate flexible integrated PDs and future optoelectronic systems.

## Conflict of Interest

The authors declare no conflict of interest.

## Author Contributions

The conceptualization of the project was performed by D.I.Z., W.H., A.F., T.J.M., and M.G.K. Resources were acquired by J.C. and Z.W. Data curation was carried out by D.I.Z, W.H., J.K., D.A.Z., F.Q., D.Y.Z., J.S.K., and Y.Y., while formal analysis was conducted by Z.X. and D.B. Supervision was managed by A.F., T.J.M., M.G.K., and L.J.L. Funds were acquired by A.F., T.J.M., M.G.K., and L.J.L. The original draft was written by D.I.Z., and the writing, reviewing, and editing of the final draft was done by A.F., T.J.M., and M.G.K.

## Supporting information



Supporting Information

## Data Availability

The data that support the findings of this study are available in the supplementary material of this article.
