# Supplementary material for: Ultra‐Flexible Pixelated Perovskite Photodetectors Enabled by Honeycomb Polymer Grids for High‐Resolution Imaging
Source: Adv Mater. 2025 Mar 17;37(17):2415068. doi: 10.1002/adma.202415068 (PMC12038531; doi:10.1002/adma.202415068)
Supplement: Supplementary file 1 — Supporting Information [file ADMA-37-2415068-s001.docx]

Copyright WILEY-VCH Verlag GmbH & Co. KGaA, 69469 Weinheim, Germany, 2023.

Supporting Information

Ultra-flexible Pixelated Perovskite Photodetectors Enabled by Honeycomb Polymer Grids for High-resolution Imaging

Ding Zheng, Zhaoqian Xie*, Wei Huang*, Dongjun Bai, Jaehyun Kim, Dan Zhao, Fei Qin, Dayong Zhang, Joon-Seok Kim, Jianhua Chen, Yao Yao, Zhi Wang, Lincoln J.Lauhon, Mercouri G Kanatzidis*, Tobin J. Marks*, Antonio Facchetti*

# Materials and Methods

Device Fabrication

*Substrate cleaning and parylene deposition.* All glass and ITO/glass substrates were cleaned with detergent, acetone, DI water and IPA, then UV-treated with a UV-Ozone cleaner (Jelight, UVO-Cleaner Model 42) for 15 min. Prior to parylene deposition, 1% Micro-90 in DI water was spin-coated on the glass substrate at 2000 rpm for 60 s to facilitate delamination. Then, a 1-μm-thick parylene-C film was deposited by chemical vapor deposition (SCS Labcoater2) and was used as an ultra-thin substrate for the flexible devices. After parylene deposition all the substrates were thermally annealed at 180 ^o^C for 1 h in air. The parylene substrate remains laminated to the rigid glass during subsequent layer depositions.

*Bottom electrode preparation.* For the 2000 PPI devices, the Cr mask layer with the NU pattern letters (50 nm) was deposited by thermal evaporation of Cr and then patterned and etched by photolithography. Next, a 200 nm SU8 (SU-8 3005, MicroChem) layer was spin-coated (8000 rpm for 60 s) for planarization and fill-in of the edges. After curing of the SU8 (95 ^o^C for 20 min), a 100 nm ITO layer was deposited by sputtering (AJA Orion Sputter System, RF sputter with no O2 injection) at room temperature, yielding the device section shown in Figure 2c.

For the 16 × 16 cross-bar devices, the same perylene substrates were used but without the Cr mask. A 100-nm-thick ITO bottom electrode was sputtered as reported above and patterned by photolithography (AZ® nLOF 2035, MicroChem) and then etched by ITO etchant (TE100, Transene) for 10 min at 45 ^o^C to form ITO bottom line-scan electrodes (Figure S9). Au connecting pads (50 nm) were fabricated by thermal evaporation, patterned by photolithography (AZ® nLOF 2035, MicroChem) and then etched by Au etchant (GE-8110, Transene) for 3 min at room temperature.

*Fabrication of polymer grid.* Honeycomb and perforated polymer grids were fabricated with the breath Figure method^[1-3]^. First, the polymer Cinnamate-cellulose (Cin-cell)^[4]^ was dissolved in chloroform:methanol (86: 14) solution at a concentration of 16 mg/mL. Next, a PVA solution (16mg/mL in water) was spin-coated at 3000 rpm for 30 s on a thin glass substrate (0.1 mm, 2.5 ×2.5 cm^2^) acting as a sacrificial layer. Then, the Cin-cell solution was spun on the PVA/glass substrate at 5000 rpm for 10 s in a humidity control box (>90% RH) to form the Cin-cell honeycomb grid. To prevent the Cin-cell grid under following solvents interaction, the cross-linkable Cin-cell grid was crosslinked by UV flood exposure (Inpro Technologies F300S) for 30s. The as-formed polymer gird coated on glass/PVA substrates are ready for transfer process onto any target substrates.

*Perovskite film and photodetector fabrication.* The perovskite photodetectors have a p-i-n architecture of structure: Parylene/ITO/PTAA/(w/wo polymer grid)-perovskite/PC60BM:PMMA/BCP/Ag. First, a PTAA^[5]^ solution (4mg/mL in toluene) was spun onto the patterned ITO/parylene/glass substrates at 4000 rpm for 30 s to form the HTL. Next, the nionic perovskite photodetector devices were fabricated by laminating the Cin-cell polymer grid to the HTL. This was achieved by first immersing the glass/ PVA/ Cin-cell grid substrates in water to delaminate the Cin-cell film, causing it to float on the water surface (See Figure S2), and then carefully transferring it to the PTAA coated substrates. The transferred films were first dried with an N2 gun and then annealed at 100 ^o^C for 10 min. Next, the glass/parylene/ITO/PTAA/Cin-cell grid substrates were transferred to a dry box (RH < 5%) to deposit the perovskite layer following the two-step spin method^[6]^. First, a PbI2 solution (220 mg/mL in DMF:DMSO = 95:5 vol) was spun on the Cin-cell layer at 5500 rpm for 40 s. Then, the films were annealed 70 ^o^C for 1 min. After cooling to the room temperature, a FAI:MABr:MACl solution (22 mg:2.2 mg: 2.2 mg in 1mL IPA) was spun on the PbI2 layer at 5500 rpm for 40 s. The resulting samples were annealed at 140 ^o^C for 15 min on a hot plate in a 30%-40% RH atmosphere, resulting in a ~250 nm thick perovskite layer embedded into the Cin-cell grid. Finally, the device was completed by the deposition of a 30 nm thick PC60BM/PMMA layer (10:1, 25 mg/mL in chlorobenzene, spin with 3000 rpm/min, annealing at 100 oC for 5 min), a 3 nm thick BCP layer (0.5 mg/mL in methanal, spin with 3000 rpm/min) and thermal evaporation of Ag top electrodes (80 nm thick) through a shadow mask. Note, for the 2000 PPI devices, the Ag electrodes (12.5 μm × 12.5 μm) were fabricated using a 2000 mesh TEM grid as the mask. As the control, the unpatterned perovskite films were fabricated as described above for the pixelated but without the steps involved in Cin-cell grid deposition.

Device Characterization

Electrical measurements for the 2000 PPI devices were carried out with an ultra-fine probe station (Signatone) and an Agilent 4155C semiconductor parameter analyzer in ambient. For the 16 × 16 cross-bar devices, measurements were carried out with an Agilent B1500. For all current mapping measurements, data were taken point-by-point manually. For incident angle-dependent light current measurement, a laser beam (LPGLV5, 1.0 mRad, 532 nm, 83mW/cm^2^) was fixed on a homemade angle turning holder to illuminate the device curvatures at different angles (Beam dimension is 0.2 mm). Note under angle dependance measurements, all 16 bottom and top electrodes are connected together separately. Noise current was measured using a noise current preamplifier and a vector-signal analyzer in dark. FOV measurements were conducted with an angle-turntable custom-made holder with a fixed laser beam (LPGLV5, 1.0 mRad, 532 nm, 83mW/cm^2^), the devices are then connected to a Agilent B1500 to record the light current for different incident angles. The on-off switch stability and long-term light soaking stability measurements were performed under the following experimental conditions: The devices were tested at a temperature of 25°C and relative humidity (RH) of 45%, without encapsulation using parylene. The on-off switching test was conducted over 28,800 seconds at a switching frequency of 2 Hz by using 532 nm and 83mW/cm^2^green laser. For the long-term light soaking test, the unencapsulated devices were stored in an aging box under the same temperature (25°C) and humidity (45%) conditions, exposed to a white LED light source with an intensity of 100 mW/cm². Light current was measured daily with an AM 1.5 light source over a period of 10 days.

Film Characterization

SEM and AFM images were recorded with a Hitachi SU8030 FE-SEM and a Bruker ICON System, respectively.

Mechanical simulations.

A finite element analysis (FEA) commercial software ABAQUS was used to model the strain distribution in the unpatterned and pixelated perovskite films on elastomer under pure bending. Four-node plane strain quadrilateral elements (CPE4R) with reduced integration and hourglass control were adopted. Convergence test of the mesh size was performed to ensure accuracy. The elastic modulus (*E*) and Poisson’s ratio (ν) of the film components are: *E*PVK=11 GPa and νPVK=0.35 for perovskite, *E*Polymer=600 MPa and νPolymer=0.35 for polymer, and *E*Parylene=2.8 GPa and νParylene=0.4 for parylene C.

**Table S1.** Basic devices properties of perovskite PDs in references.

|  | Materials | *D**  (Jones) | Pixel sizes  (μm) | Noise current  (mA/Hz^-1/2^) | Rise/fall time  (μs) | Bending radius  (μm) | FOV  （^o^） | Shape |
| --- | --- | --- | --- | --- | --- | --- | --- | --- |
| This work | (FAPbI_3_)_0.97_(MAPbBr_3_)_0.03_ | 3.91×10^13^ | 1.5 | 1.37×10^-13^ | 3.94/4.81 | 5 | 216 | Planar/ hemisphere |
| Ref.^[7]^ | FAPbI_3_ | ~2.7×10^9^ | 0.5 | - | 21800/29900 | Rigid | 130 | hemisphere |
| Ref.^[8]^ | CH_3_NH_3_PBI_3_ | 1.22×10^13^ | - | - | - | - | - | Planar |
| Ref.^[9]^ | Cs_3_Bi_2_Br_9_ | 3.39×10^11^ | - | - | 570/580 | Rigid | - | Planar |
| Ref.^[10]^ | CsBi_3_I_10_ | - | - | 10^-13^ | - | Rigid | - | Planar |
| Ref.^[11]^ | PEA_0.15_FA_0.85_SnI_3_ | 8.29×10^11^ | - | - | 0.78/0.83 | Rigid | - | Planar |
| Ref.^[12]^ | DMABI | 3.18×10^13^ | - | - | 149000/  252000 | Rigid | - | Planar |
| Ref.^[13]^ | (BA)_2_MAPb_2_Br_7_-MAPbBr_3_ | 3.90×10^13^ | - | - | - | Rigid | - | Planar |
| Ref.^[14]^ | Cs_0.05_[(FA)_0.83_(MA)_0.17_]_0.95_Pb(I_0.9_Br_0.1_)_3_ | 4.70×10^12^ | - | 1.00×10^-14^ | 46/113 | Rigid | - | Planar |
| Ref.^[15]^ | (C_6_H_5_(CH_2_)_2_NH_3_)_2_PbI_4_ | 1.21×10^13^ |  | - | 78/105 | - | - | Planar |
| Ref.^[16]^ | CsPbBr_3_ | 1.25×10^13^ |  | 10^-15^ | 1.41/2.06 | Rigid | - | Planar |
| Ref.^[17]^ | CsPbBr3 | 3.94×10^13^ | 500 | - | 800/650 | 20 | - | Planar/  hemisphere |


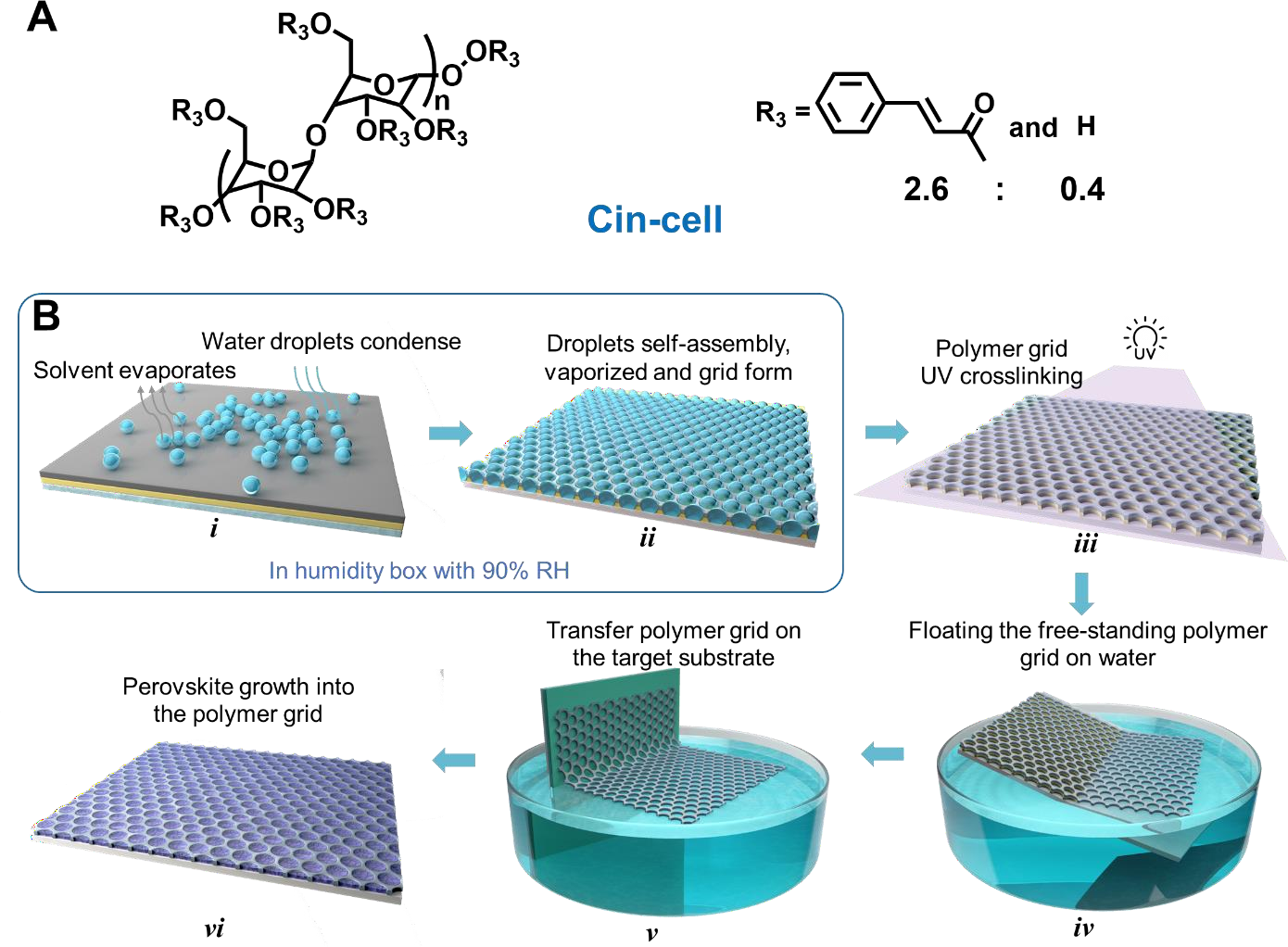


**Figure S1.** (a) polymer structure of photocurable Cin-cell. (b) Fabrication process for pixelated perovskite photodetector (PD): i-ii). Water droplets condense, self-assembly and sink into the cellulose poly and form the honeycomb structure; iii). Crosslinking the cellulose under UV light; iv): floating and delaminating the free- standing cellulose film on water; v): pick up the perforated cellulose film on any target substrates;vi): Spin-coating the perovskite solution to embed the perovskite materials in the cellulose grid.


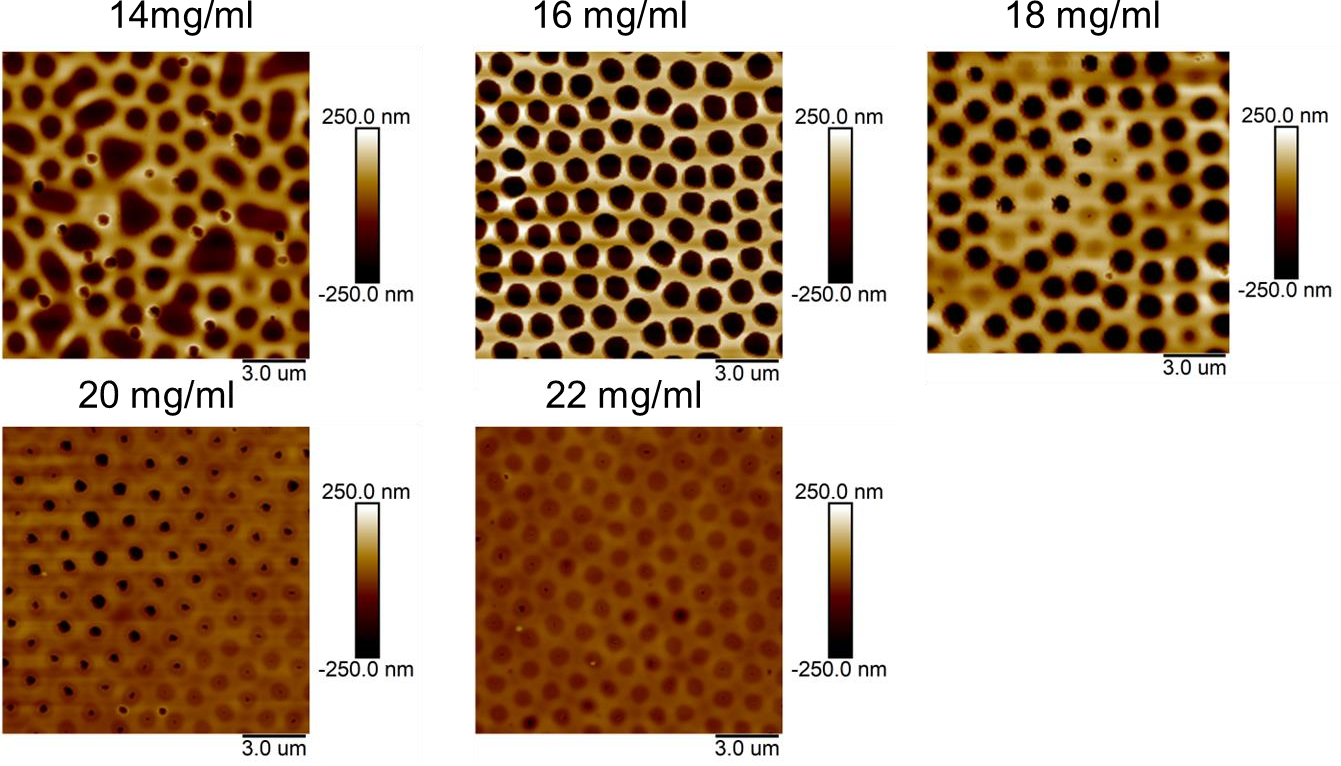


**Figure S2.** AFM images of Cin-Cell grid films fabricated by the Breath-Figure method using different polymer concentrations. After several trials, the best concentration was found to be 16 mg/mL


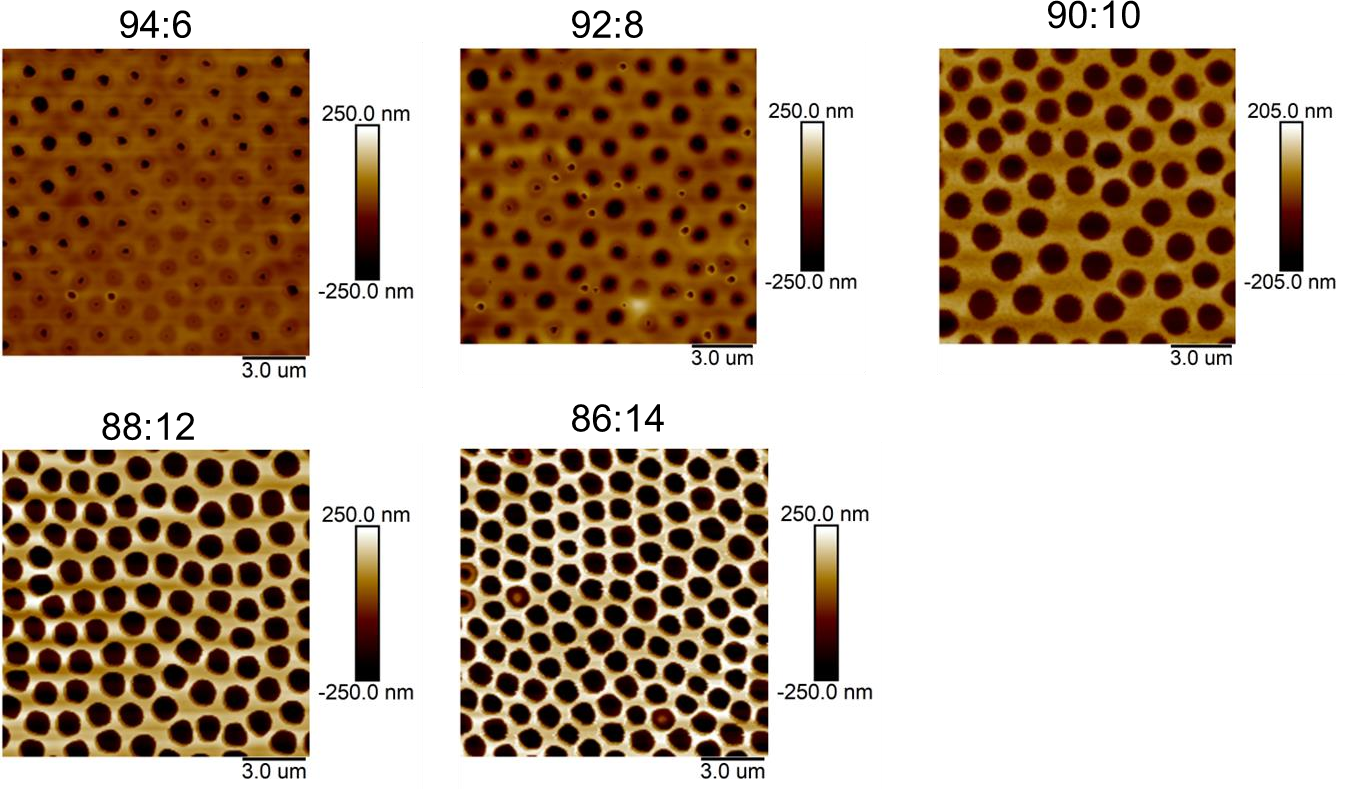


**Figure S3.** AFM images of Cin-Cell grid fabricated by the Breath-Figure method with different chloroform:methanol volume ratios. The optimal ratio for this work is 86:14 vol/vol but this result demonstrates that different resolutions are possible widening applications.


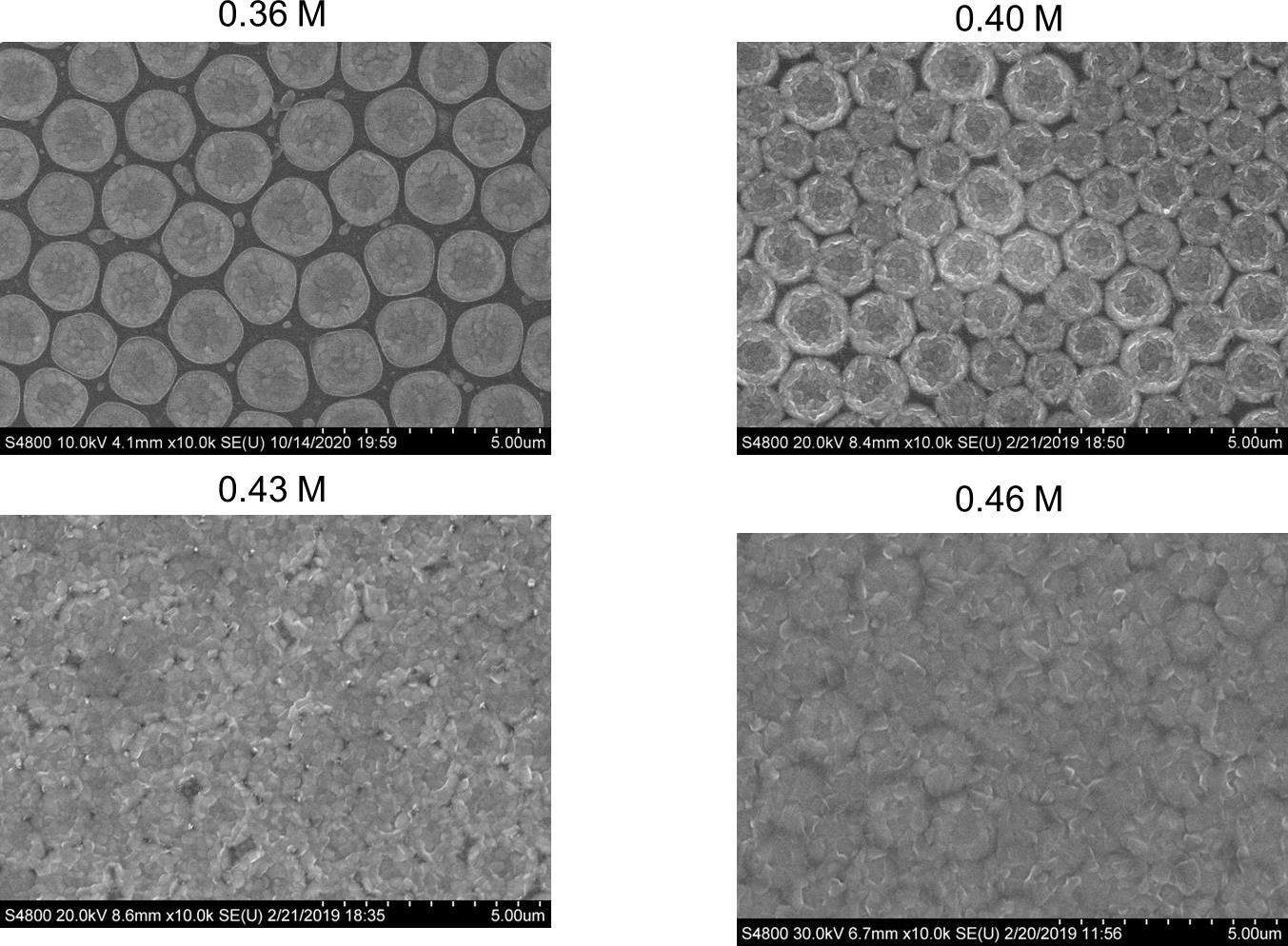


**Figure S4.** SEM images of perovskite films deposited on Cin-Cell grids using different concentrations of the perovskite precursors. The optimal concentration of the perovskite precursors is 0.36 M, which provides a delicate balance between perovskite formation and grid integrity.


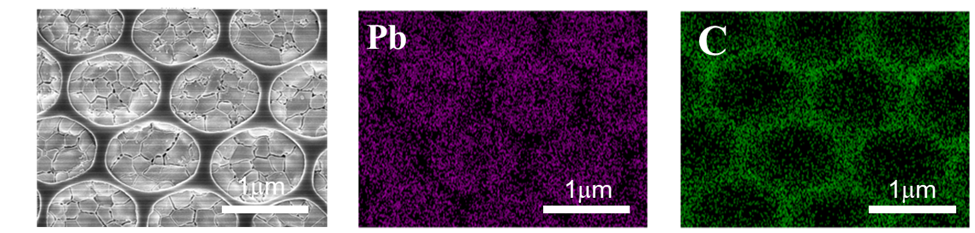


**Figure S5.** SEM and EDS analysis of a perovskite film embedded in a Cin-Cell grid.

**Figure S6.** Steady-state absorption spectra of unpatterned and pixelated perovskite films.

**Figure S7.** Steady-state transmittance spectra of unpatterned and pixelated perovskite films.

**Figure S8.** XRD spectra of unpatterned and pixelated perovskite films.

**Figure S9.** Steady-state PL spectra of unpatterned and pixelated perovskite films.


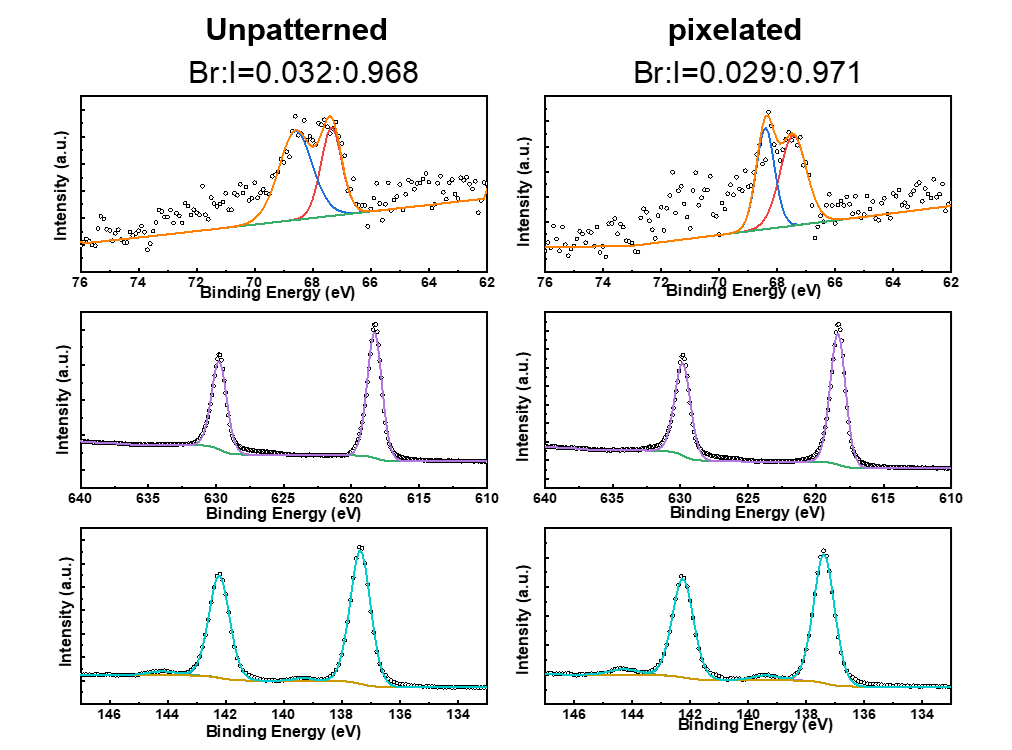


**Figure S10.** XPS spectra of Br3d (top), I3d (middle), and Pb4f (bottom) of unpatterned and pixelated perovskite films.


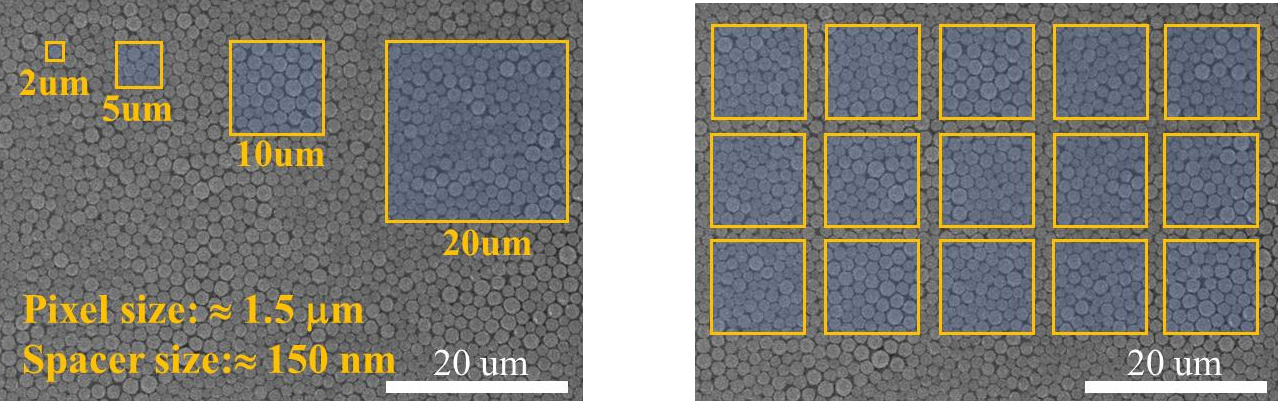


**Figure S11.** Scanning electron microscopy (SEM) images of the pixelated perovskite films and a schematic diagram illustrating the potential resolution of the arrays. Note, the resolution of the corresponding devices is determined by both the size of the perovskite pixels and the electrodes. In the present study, the minimum pixel size is approximately 1.5 μm, indicating that the highest achievable resolution of the pixelated perovskite PD is ~16,500 pixels per inch (PPI) if the electrode size matches that of the active pixel. For this work, we employed an electrode size of 10x10 μm^2^, thus affording a resolution of ~ 2000 PPI.


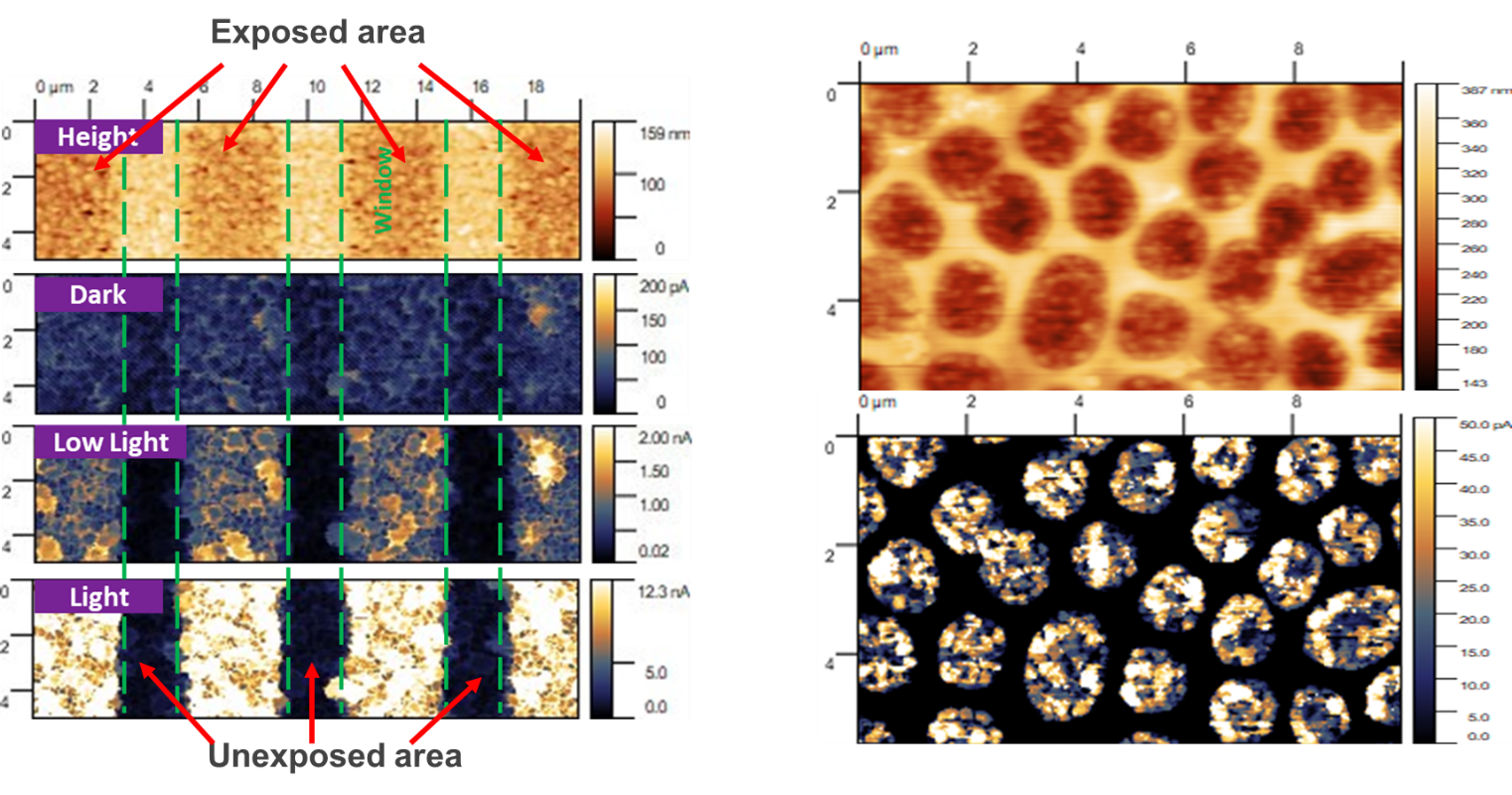


**Figure S12**. Conducting-AFM current mapping of an unpatterned perovskite film (left) and a pixelated perovskite film within the Cin-Cell grid (right).
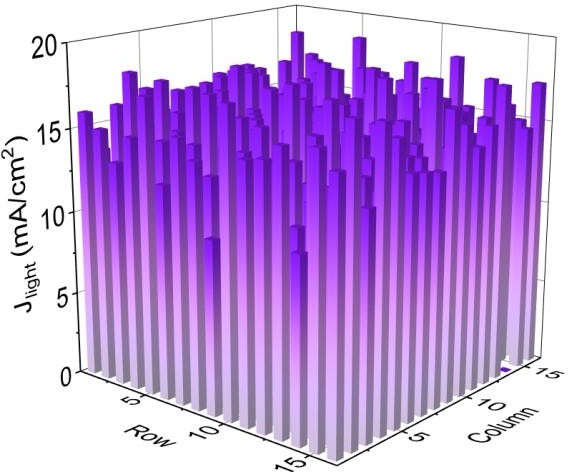


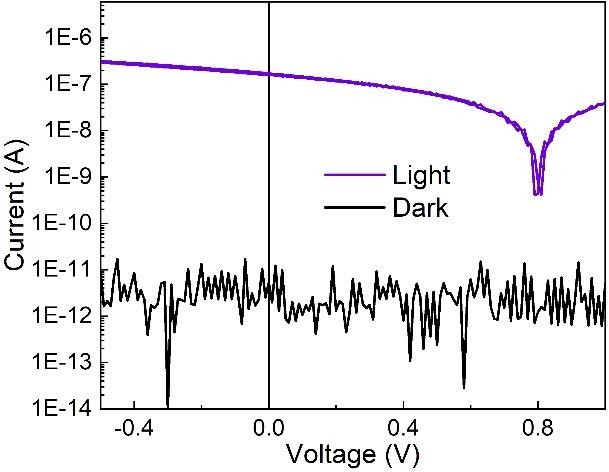


**Figure S13**. Current-voltage characteristics under AM 1.5 G illumination and in the dark of an individual pixel for the unpatterned perovskite photodiode array. (f) Light current density distribution at -0.3 V of a 16 × 16 unpatterned perovskite photodiode arrays.


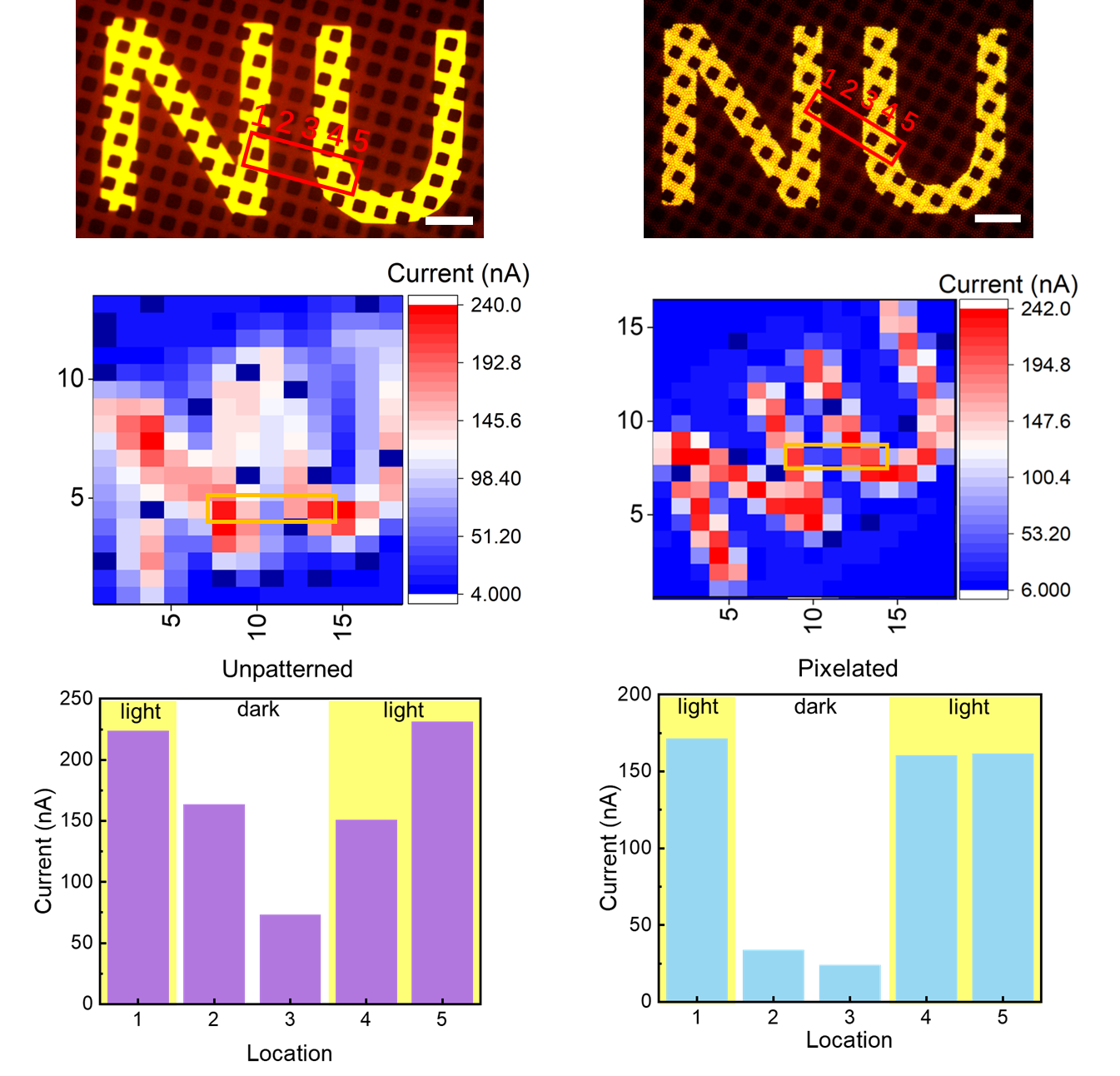


**Figure S14**. The current extracted from locations on both unpatterned and pixelated devices. Locations 1, 4, and 5 correspond to the light pixels, while locations 2 and 3 represent the dark pixels.


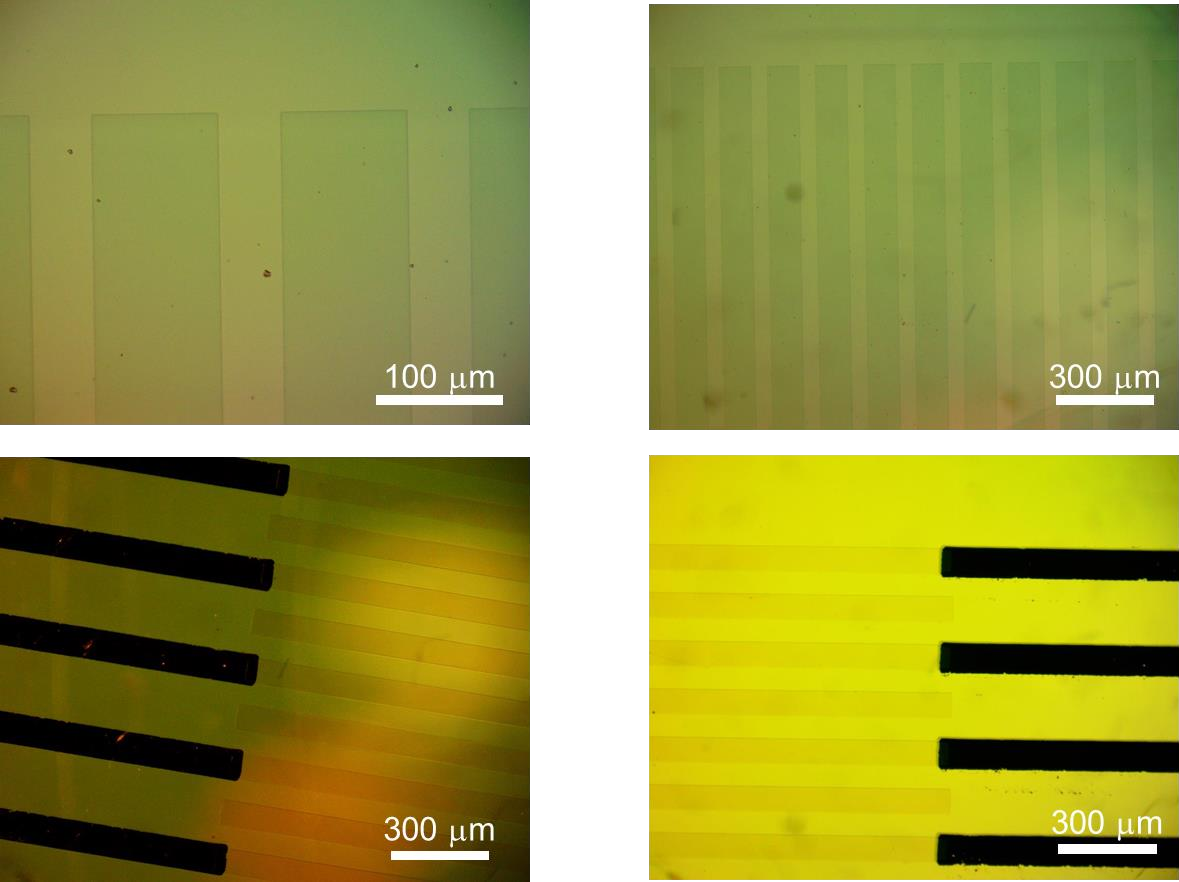


**Figure S15.** Microscope images of bottom ITO electrodes with Au contacts for a 16 × 16 cross-bar array.


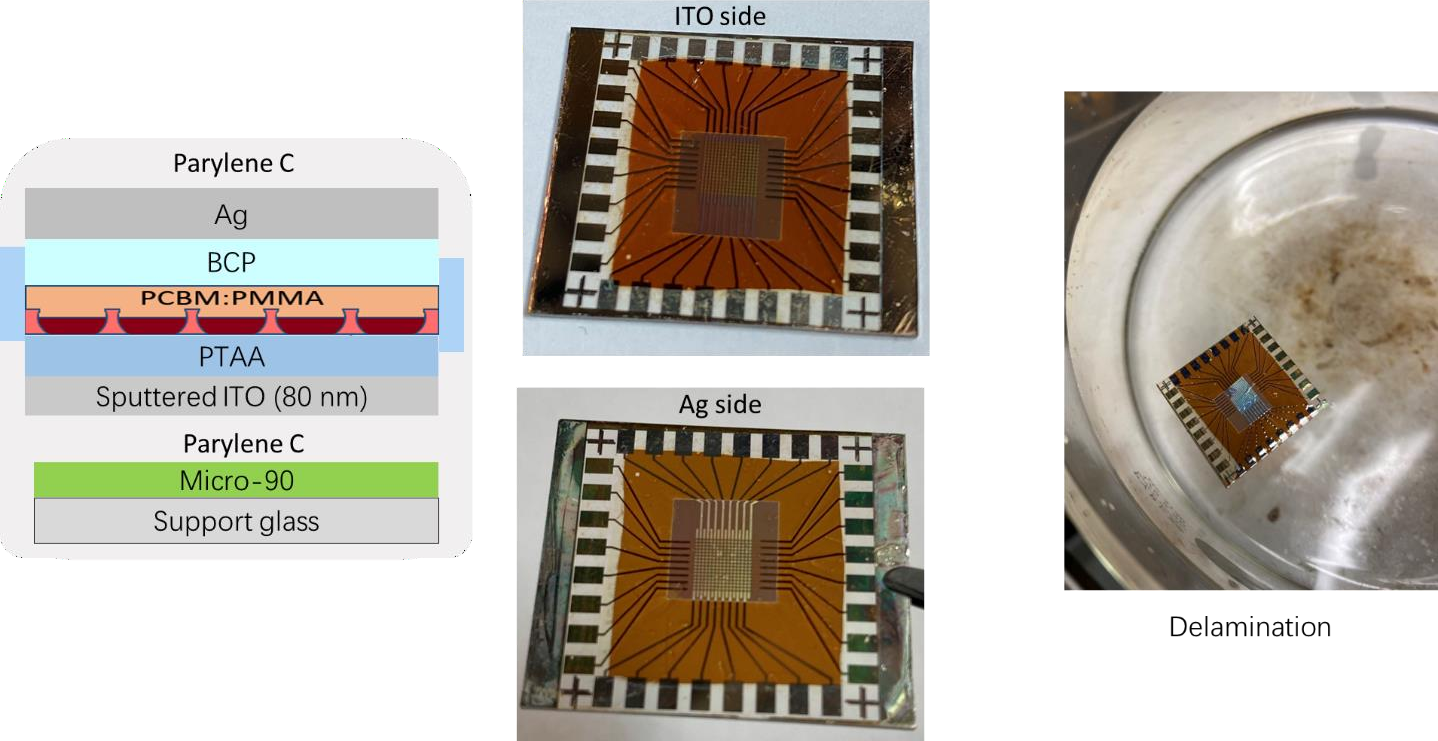


**Figure S16**. Device architecture is depicted on the left, with an image in the middle showing a laminated, double-sealed 16 x 16 cross-bar array encapsulated between two parylene films. On the right, an image of the delaminated device floating on water demonstrates the waterproof properties,attribute to the double-sealed parylene structure.


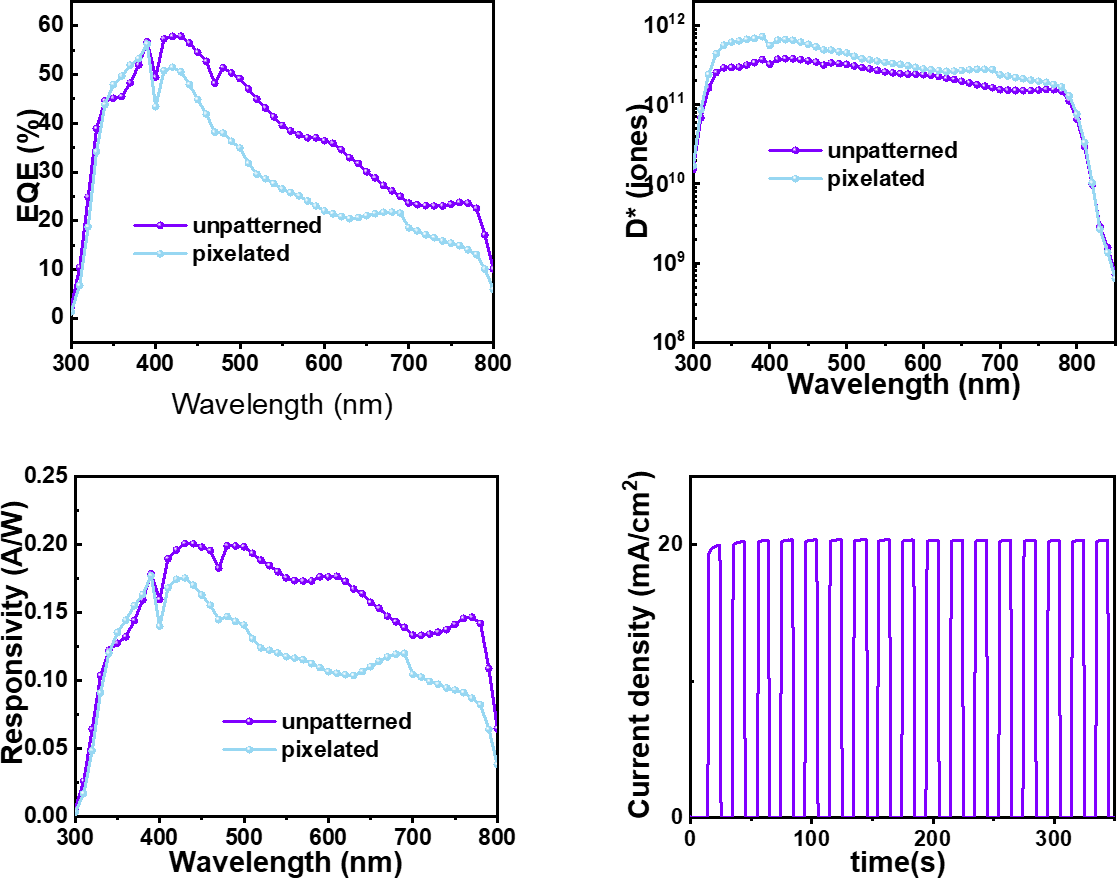


**Figure S17**. EQE, wavelength dependent detectivity, responsivity and on-off current cycling test of a 16 ×16 array based on unpatterned and pixelated perovskite films.


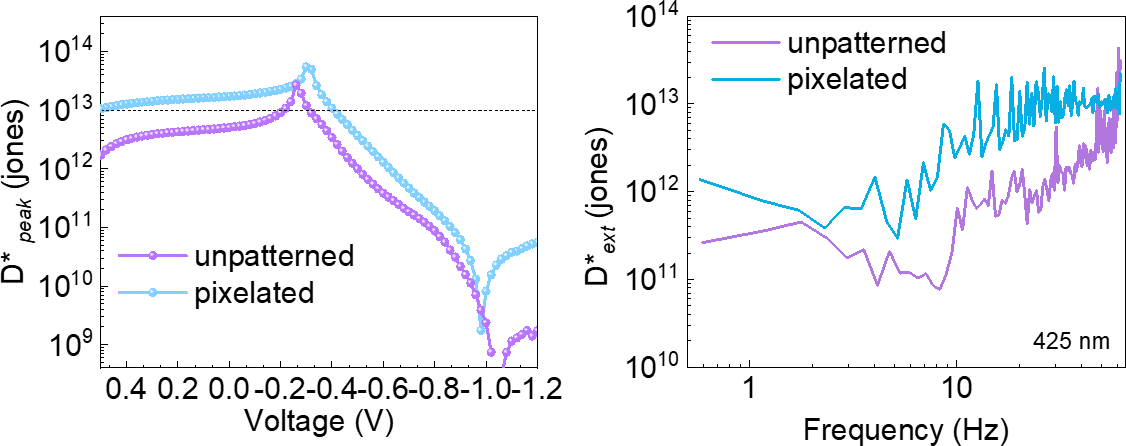


**Figure S18.** Calculated D**peak* (left) and D**ext* (right) of 16 ×16 arrays based on unpatterned and pixelated perovskite films.

To calculate D**peak*, the following equation was used^[18]^:

*R =Iph/P, D*peak*

Where *Iph* is the photocurrent (Iph=Ilight−Idark),P is the light power, R was extracted from the EQE(λ = 425 nm).

To calculate D**ext*, the following equation was used^[19]^:

D**ext* = (A⋅B)^1/2^/NEP

Where A is Ais the illuminated area (channel W×L = 100 μm × 100 μm), B is the spectral bandwidth, and NEP is the noise equivalent power = Noise current/R.


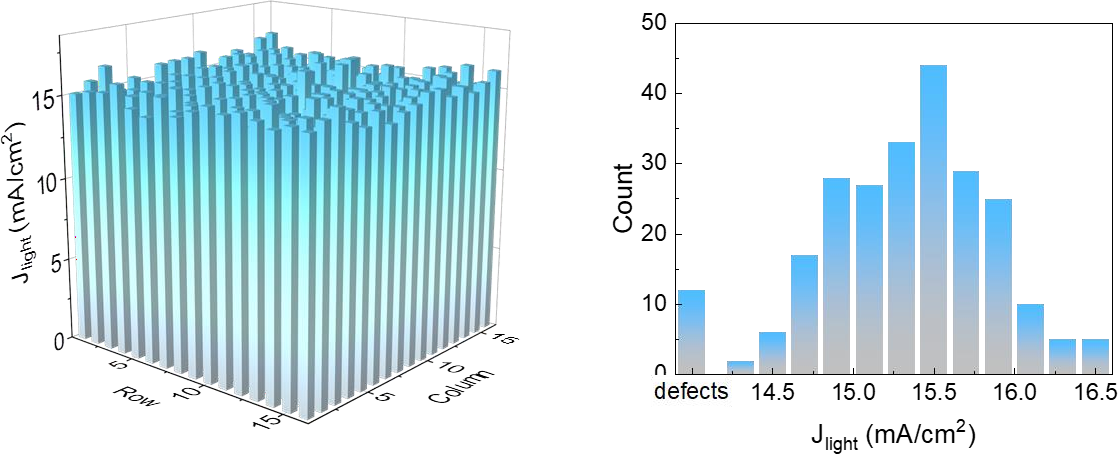


**Figure S19**. Left: light current density distribution of a 16 × 16 pixelated perovskite PDs array under -0.3 V bias. Right: Statistical distribution histograms of light current density for 256 pixels including 12 defects.


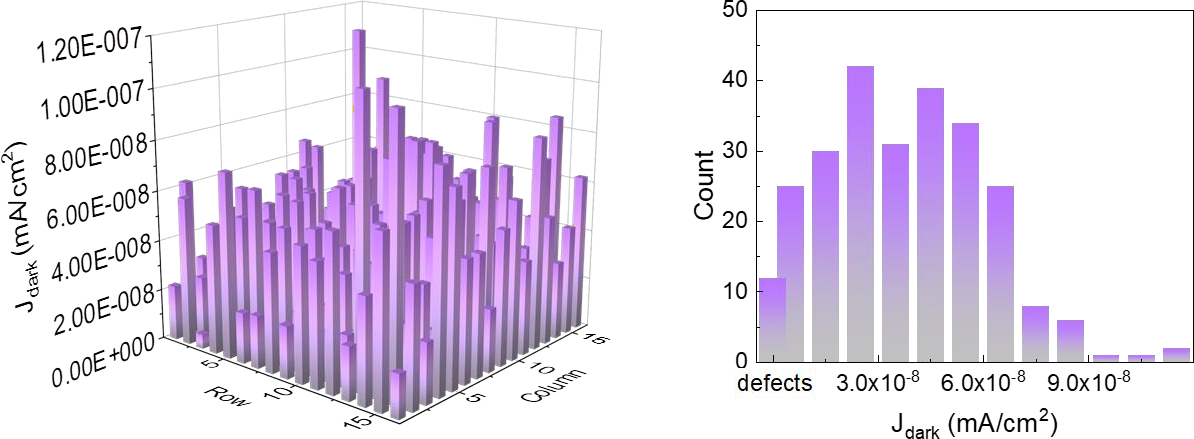


**Figure S20**. Left: Dark current density distribution of a 16 × 16 pixelated perovskite PDs array under -0.3 V bias. Right:Statistical distribution histograms of dark current density for 256 pixels including 12 defects.


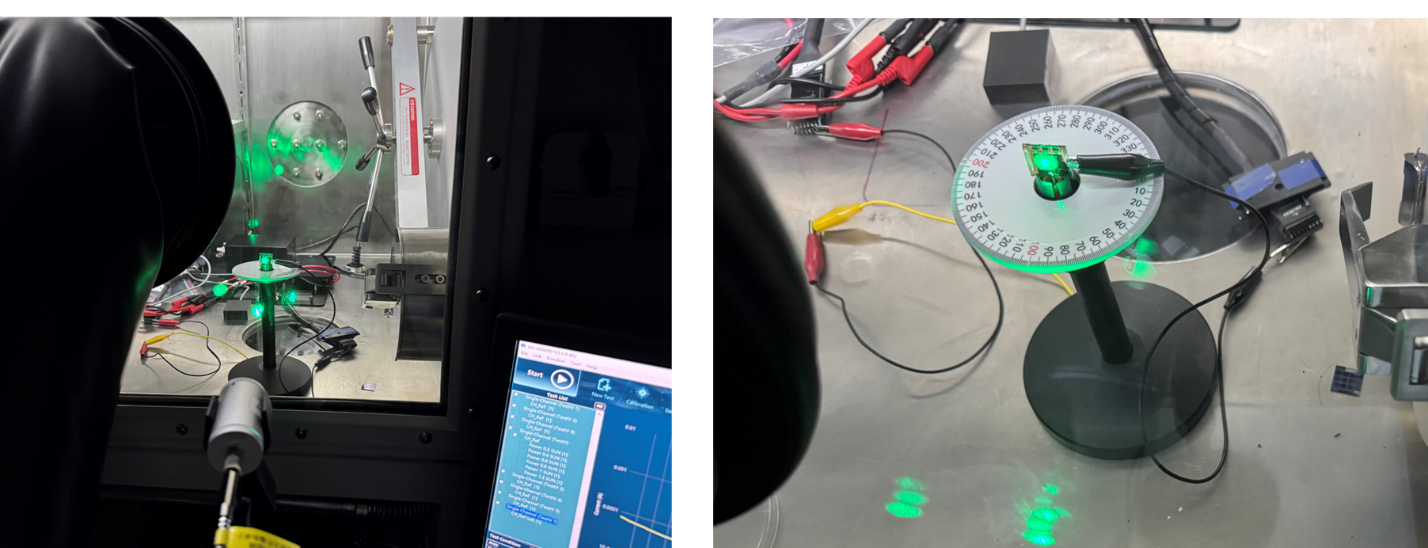


**Figure S21.** FOV measurement setup for the perovskite PDs. A custom-made holder to precisely adjust the incident angle of the light source. The holder is equipped with a fixed laser beam that can be rotated to vary the angle of incidence. The devices are then connected to a standard I-V measurement setup to record the light current at different incident angles.

150


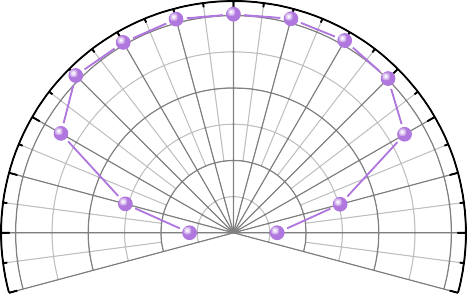
100

50

Current (μA)

0

50

100

150

120

135

150

165

180

195

105 90 75

60

45

30

15

0

-15

**Figure S22**. Incident angle dependent light current of a planar 16 × 16 cross-bar array based on an

unpatterned perovskite film.

**Figure S23**. On-off operation stability of the pixelated perovskite PD under stress conditions of T = 25°C and RH = 45%, without encapsulation.

**Figure S24**. Long-term light soaking stability of pixelated perovskite PD under stress conditions of T = 25°C and RH = 45%, without encapsulation.


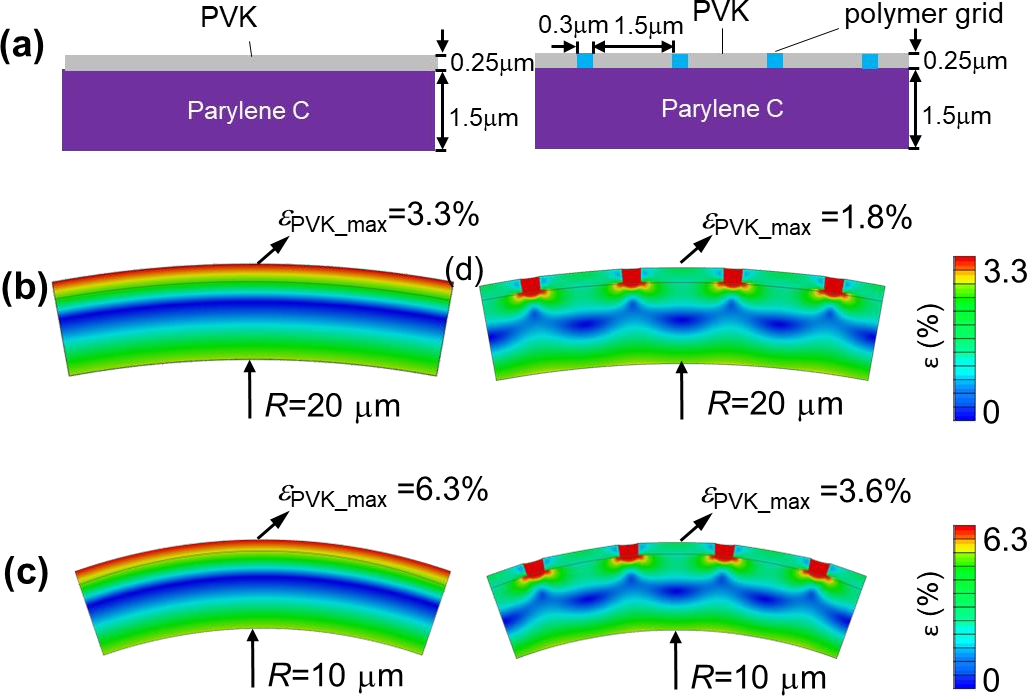


**Figure S25.** Pure bending at a radius *R* of unpatterned (left) and pixelated (right)) perovskite (PVK) films on parylene C. The distributions of maximum principal strain ɛ under pure bending of (a) *R*= 0 μm (unstrained films). (b) *R*= 20 μm, (c) *R*= 10 μm, where *ε*Perovskite_max is the maximum ɛ in perovskite materials.

# References:

[1] J. Chen, W. Huang, D. Zheng, Z. Xie, X. Zhuang, D. Zhao, Y. Chen, N. Su, H. Chen, R. M. Pankow, Z. Gao, J. Yu, X. Guo, Y. Cheng, J. Strzalka, X. Yu, T. J. Marks, A. Facchetti, Nat. Mater. 2022, 21, 564.

[2] X. Zhang, B. Wang, L. Huang, W. Huang, Z. Wang, W. Zhu, Y. Chen, Y. Mao, A. Facchetti, T. J. Marks, Science Advances, 6, eaaz1042.

[3] V.-T. Bui, S. H. Ko, H.-S. Choi, ACS Appl. Mater. Interfaces 2015, 7, 10541.

[4] Z. Wang, X. Zhuang, Y. Chen, B. Wang, J. Yu, W. Huang, T. J. Marks, A. Facchetti, Chem. Mater. 2019, 31, 7608.

[5] Q. Wang, C. Bi, J. Huang, Nano Energy 2015, 15, 275.

[6] Q. Jiang, L. Zhang, H. Wang, X. Yang, J. Meng, H. Liu, Z. Yin, J. Wu, X. Zhang, J. You, Nat. Energy 2016, 2, 16177.

[7] L. Gu, S. Poddar, Y. Lin, Z. Long, D. Zhang, Q. Zhang, L. Shu, X. Qiu, M. Kam, A. Javey, Z. Fan, Nature 2020, 581, 278.

[8] S.-F. Leung, K.-T. Ho, P.-K. Kung, V. K. S. Hsiao, H. N. Alshareef, Z. L. Wang, J.-H. He, Advanced Materials 2018, 30, 1704611.

[9] Z. Ji, Y. Liu, W. Li, C. Zhao, W. Mai, Science Bulletin 2020, 65, 1371.

[10] Z. Ji, Y. Liu, M. Yao, Z. Zhang, J. Zhong, W. Mai, Advanced Functional Materials 2021, 31, 2104320.

[11] A. Ajayakumar, A. Sławek, C. Muthu, A. V. Dev, N. K. Shajan, A. Ajith, K. Szaciłowski, C. Vijayakumar, Advanced Materials 2024, n/a, 2411332.

[12] X. Fan, E. Hong, P. Wang, X. Fang, Advanced Functional Materials 2024, n/a, 2415491.

[13] D. Nodari, L. J. F. Hart, O. J. Sandberg, F. Furlan, E. Angela, J. Panidi, Z. Qiao, M. A. McLachlan, P. R. F. Barnes, J. R. Durrant, A. Ardalan, N. Gasparini, Advanced Materials 2024, 36, 2401206.

[14] W. Wang, W. Tian, F. Chen, J. Wang, W. Zhai, L. Li, Advanced Materials 2024, 36, 2404968.

[15] S. Zhang, Y. Ge, X. Qin, X. Wang, T. Tao, L. Yu, X. Song, Y. Jiang, C. Xia, Advanced Functional Materials 2024, n/a, 2418968.

[16] H. Zhu, H. Chen, J. Fei, Y. Deng, T. Yang, P. Chen, Y. Liang, Y. Cai, L. Zhu, Z. Huang, Nano Energy 2024, 125, 109513.

[17] W. Wu, X. Han, J. Li, X. Wang, Y. Zhang, Z. Huo, Q. Chen, X. Sun, Z. Xu, Y. Tan, C. Pan, A. Pan, Advanced Materials 2021, 33, 2006006.

[18] C. Fuentes-Hernandez, W.-F. Chou, T. M. Khan, L. Diniz, J. Lukens, F. A. Larrain, V. A. Rodriguez-Toro, B. Kippelen, Science 2020, 370, 698.

[19] J. Kim, S.-M. Kwon, Y. K. Kang, Y.-H. Kim, M.-J. Lee, K. Han, A. Facchetti, M.-G. Kim, S. K. Park, Science Advances, 5, eaax8801.
